# A Rare Case of Concomitant Maxilla and Mandible Brown Tumours, Papillary Thyroid Carcinoma, Parathyroid Adenoma, and* Osteitis Fibrosa Cystica*

**DOI:** 10.1155/2016/5320298

**Published:** 2016-01-03

**Authors:** Thaís Borguezan Nunes, Sheyla Batista Bologna, Andréa Lusvarghi Witzel, Marcello Menta Simonsen Nico, Silvia Vanessa Lourenço

**Affiliations:** ^1^Department of Stomatology, School of Dentistry, University of São Paulo, Avenida Professor Lineu Prestes 2227, Cidade Universitária, 05508-000 São Paulo, SP, Brazil; ^2^Department of Dermatology, Medical School, University of São Paulo, Avenida Dr. Enéas de Carvalho 255, 3o Andar, Sala 3068, 05403-000 São Paulo, SP, Brazil

## Abstract

*Objective.* The brown tumour of hyperparathyroidism is a result of a metabolic disorder caused by primary hyperparathyroidism.* Report*. We described a case of a 37-year-old female patient presenting bimaxillary intraoral lesions and swelling in the neck. Incisional biopsy of the oral lesion was performed and histopathological examination revealed a central giant cell lesion composed by intense haemorrhagic exudate, abundant presence of giant cells, and areas with hemosiderin pigment. The patient also presented high levels of serum calcium and parathyroid hormone, hyperfunctioning parathyroid tissue, bilateral parenchymal nephropathy, and densitometry lower than expected, showing an advanced stage of* osteitis fibrosa cystica*. Synchronous parathyroid adenoma and papillary thyroid carcinoma were confirmed by imaging exams and histopathologically.* Conclusion.* The composition of all the clinical, pathological, and imaging findings led to the final diagnosis of brown tumour of hyperparathyroidism. The occurrence of parathyroid adenoma, papillary thyroid carcinoma, and brown tumours of hyperparathyroidism in their late stage (*osteitis fibrosa cystica*) associated with oral brown tumours involving the mandible and maxilla is extremely rare.

## 1. Introduction

Severe parathyroid bone disease is rare.* Osteitis fibrosa cystica* is the end-stage manifestation of primary hyperparathyroidism. The bone lesions are the result of a metabolic disorder caused by the altered parathyroid, mainly due to adenomas in 85% of the patients [1]; the overproduction of parathyroid hormones (PTH) causes imbalance in osteoclast activity resulting in bone destruction [2]. Histologically, the lesions are characterised by the presence of osteoclasts associated with areas of irregular bone resorption, which is replaced by fibrous vascularised tissue (fibrous cysts) and osteoclast-like giant cells. Accumulation of red blood cells and their pigments give a reddish/brown hue to the lesions, hence the name “brown tumour” [3].

Brown tumours of hyperparathyroidism are infrequent today due to improved medical technology that enables early diagnoses and correction of PTH levels [4]. The classical bone lesions are most frequently found in long bones, pelvis, and ribs. Facial involvement is only exceptional and, when present, usually involves the mandible. The occurrence of parathyroid adenoma, papillary thyroid carcinoma, and brown tumours of hyperparathyroidism involving the mandible and maxilla is extremely rare [5, 6].

We report herein a very rare case of concomitant maxilla and mandible brown tumours, thyroid carcinoma, parathyroid adenoma, and* osteitis fibrosa cystica.*

## 2. Case Report

A 37-year-old woman presented to the Clinics of the Dental School, University of São Paulo, complaining of oral pain and facial deformity. The patient reported bleeding and painful oral lesions over the last 6 months.

**Figure 1:**
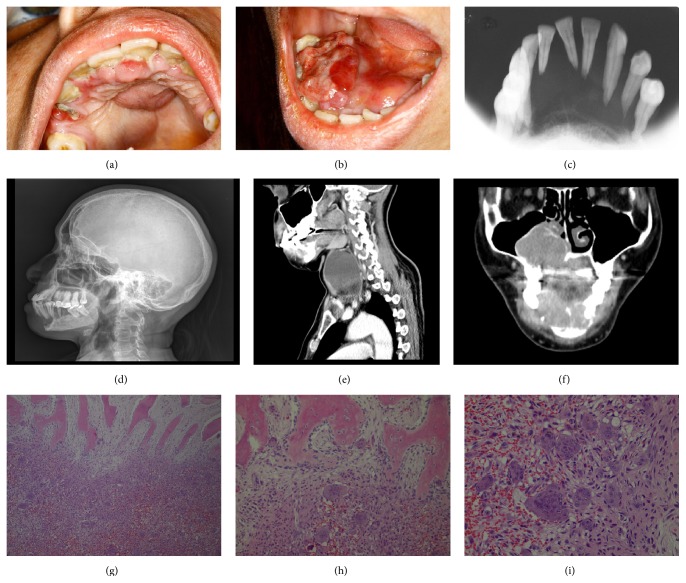
(a)-(b) Clinical aspects of the maxilla and mandible lesions: haemorrhagic tumour masses involving the vestibular and lingual/palatal gingiva. (c) Radiographic aspects of the occlusal X-ray: large radiolucent area involving the teeth roots, with resorption (floating teeth). (d) Radiographic aspects of the lateral skull X-ray: evidence of an extensive bone loss in the mandible, resulting in a floating teeth appearance. (e) Sagittal CT scan image: expansive lesion with anterior displacement of the thyroid lobe and posterior contact with prevertebral muscles. (f) Coronal CT scan image: expansive lesions with central soft parts components and involvement of medial right maxillary sinus wall, alveolar and palatine processes up to the floor of the nasal fossa; the lesion is also seen in the right mandibular ramus and on the mentonian region, with involvement of the anterior teeth. (g)–(i) Histopathological aspects of the mandible lesion: the mass of multinucleated giant cells permeated by haemorrhagic infiltration next to the mandibular compact bone trabeculae (g); detail of multinucleated giant cells, fibroblasts, blood cells, and inflammatory infiltrate close to compact bone trabeculae (h); multinucleated giant cells and blood cells that compose the mandibular lesion (i). Haematoxylin and eosin, original magnification ×40, ×200, and ×400, respectively.

The physical examination revealed a consistent cervical mass on the left side of the neck. Intraoral inspection showed an exophytic mass extending from the right mandibular lateral incisor to the molars. The associated teeth were mobile and enclosed by the tissue growth. In the maxilla, a swelling was seen across the palatal midline, extending from anterior region to posterior right side. Both maxilla and mandible lesions were soft in consistency and tender on palpation (Figures 1(a)-1(b)).

The patient's past medical and family history were noncontributory. Due to the severity of the case, the patient was referred to the Clinics Hospital, Medical School, University of São Paulo (HCFMUSP), for systemic investigation and medical management.

The X-rays images revealed an ill-defined osteolytic lesion causing expansion of the affected mandible, loss of lamina dura, and roots resorption of the anterior teeth (Figures 1(c)-1(d)). Computed Tomography (CT) was used to scan sites suspected to be affected by the brown tumours. Bone rarefaction focuses were also observed in the proximal phalanx of the third toe and the ribs. In the cervical region a massive septated cystic-solid lesion was located posteriorly to the left thyroid lobe besides the presence of thyroid nodules (Figures 1(e)-1(f)).

The bone densitometry values were below the expected limits for the patient's age and parathyroid scintigraphy showed hyperfunctioning parathyroid tissue on the left side.

An incisional biopsy was performed in the mandible lesion and histopathological examination was consistent with central giant cell lesions, composed of intense hemorrhagic exudate, abundant presence of multinucleated giant cells, and areas with haemosiderin pigment (Figures 1(g)–1(i)).

The biochemical investigations were carried out and showed marked hypercalcaemia (15.70 mg/dL, range 8.60–10.20 mg/dL) and an elevated parathyroid hormone (PTH) concentration (1472.00 pg/mL, range 16–87 pg/mL) in the blood.

The patient had partial thyroidectomy and parathyroidectomy on the left side. The PTH concentration taken 1 hour after the adenoma had been removed confirmed a level within the normal range. The diagnosis of papillary thyroid carcinoma and parathyroid adenoma was confirmed histologically (serial paraffin sections).

The serum calcium and inorganic phosphorus levels were estimated daily after surgery. The postoperative hypocalcemia was administered by calcium carbonate orally in addition to vitamin D until stable levels. Thyroid hormone replacement therapy was instituted.

At 6-month follow-up, there was only a modest improvement of oral pain, but the oral lesions had remained. The patient interrupted her clinical follow-up despite the efforts of the medical team.

## 3. Discussion

The occurrence of brown tumours in both jaws concomitant with the presence of hyperparathyroidism, parathyroid adenoma, and synchronous papillary thyroid carcinoma is very rare according to the literature [7, 8]. The brown tumour of hyperparathyroidism results from a metabolic disorder that affects long bones, ribs, and pelvis. The disease is most common in adults aged 50 years and over and affects 3 females to 1 male [4]. The facial involvement, though rare, manifests itself mainly in the mandible [3].

There is a great variation in the clinical expression of hyperparathyroidism. Hyperparathyroidism may be caused primarily by excessive secretion of PTH or secondarily due to kidney disease. The resultant hypercalcemia causes various metabolic problems, such as kidney stones, gastrointestinal disturbances, and muscular weakness [1, 2]. Our patient was below the average age and due to a very low grade of educational instruction, she did not seek treatment until she presented severe deformities and metabolic disorders that impacted her life quality. Additionally, the patient reported that she was diagnosed with a “thyroid cyst” two years before, but no treatment was performed due to difficulties in assessing public health system hospitals.

Radiographically, the present case was similar to those previously reported by other authors [9]. The progressive loss of bone mineral content due to the increased PTH secretion may produce radiolucent bone lesions of ill-defined margins, as those that were seen in our patient.

The destructive bone lesions of brown tumour show similar histological features to those viewed in central giant cell lesions, aneurysmal bone cyst, and cherubism and these are considered as differential diagnosis [4, 7]. The histopathological examination was consistent with a central giant cell lesion, but the composition of all the clinical, pathological, and imaging findings enabled us to reach the final diagnosis of brown tumour of hyperparathyroidism.

The pathogenesis of this disease is caused by the marked increase in osteoclastic bone resorption followed by replacement of marrow spaces with fibrovascular tissue and increased formation of osteoid. The cystic spaces are populated with clusters of giant cells as well as with hemosiderin-filled macrophages and plump fibroblasts. Clinically, these areas appear as red-brown friable mass, hence the term brown tumour [3, 10].

Brown tumours are the late manifestation of severe hyperparathyroidism; this clinical situation is also known as* osteitis fibrosa cystica*. Occasionally,* osteitis fibrosa cystica* can be mistaken for a malignant lesion, even though it is reactive and clearly nonneoplastic [8].

Primary hyperparathyroidism is characterized by hypersecretion of PTH, which is caused by adenomas in 85% of all cases. The presence of brown tumours is recognized in less than 5% of these cases [1].

According to the literature, concomitant thyroid disease is present in 3.1% of patients with primary hyperparathyroidism [11], but the reason of this association remains unclear. Although this situation is unusual, the diagnosis of coexisting diseases should be considered. The investigation of the thyroid is recommended to identify the presence of nodules. They also must be removed during parathyroid surgical approach in patients with primary hyperparathyroidism [5, 12]. In fact, our patient was diagnosed with parathyroid adenoma and papillary thyroid carcinoma. The early identification of cancer and appropriate resection of the affected glands quickly permitted the metabolic control, as noted in the assessment of serum levels of inorganic phosphorus and PTH after the surgery.

It was not possible to evaluate whether surgical excision of parathyroid adenoma and papillary thyroid carcinoma allowed the regression of oral lesions in our patient. After 6-month follow-up, she showed discrete improvement in pain, but she did not perform imaging tests that were previously requested for bone evaluation, and after this appointment she interrupted the treatment.

Some authors report that the excision of parathyroid lesions results in controlled serum levels leading to a gradual decrease in the maxillary tumours; therefore, treatment is directed to the correction of hyperparathyroidism instead of any local intervention in bone affected by brown tumours. Once hormonal dysfunction is corrected, the bones tend to mineralize completely, resulting in the regression of the osteolytic lesions [13, 14]. Brown tumours should only be removed if they persist even after removal of parathyroid adenoma [7], when functional problems are detected [1], or if tumours are too large [15]. Although the regression of brown tumours can take several months, conservative management can avoid more invasive procedure in facial bones, preventing further deformities [14].

## 4. Conclusion

The composition of all the clinical, pathological, and imaging findings led to the final diagnosis of brown tumour of hyperparathyroidism. The occurrence of parathyroid adenoma, papillary thyroid carcinoma, and brown tumours of hyperparathyroidism in the last stage (*osteitis fibrosa cystica*) with oral manifestation involving the mandible and maxilla is extremely rare. The appropriate resection of the affected glands leads to the metabolic control of serum levels of calcium and PTH. Exuberant cases, as the one reported, still occur in developing countries due to poor and inefficient governmental health and education policies.
